# Reply to Bernardo-Filho et al. Comment on “Chwalik-Pilszyk et al. Application of Polyurethane Foam as a Material for Reducing Vibration of Wheelchair User. *Materials* 2025, *18*, 1280”

**DOI:** 10.3390/ma18225199

**Published:** 2025-11-17

**Authors:** Gabriela Chwalik-Pilszyk, David Cirkl, Marek S. Kozien

**Affiliations:** 1 Faculty of Mechanical Engineering, Cracow University of Technology, 31864 Cracow, Poland; marek.kozien@pk.edu.pl; 2 Faculty of Mechanical Engineering, Technical University of Liberec, 46117 Liberec, Czech Republic; david.cirkl@tul.cz

We would like to thank Bernardo-Filho et al. for their insightful and valuable comments [1], which allow us to take a broader and more clinically relevant view of the problem under analysis.

In response to your first question, the polyurethane cushion can indeed serve a dual function. It improves sitting posture by providing better pressure distribution and enhancing user comfort, while, at the same time, it reduces the vibrations transmitted to wheelchair users. Similar observations have also been reported in the literature, which further supports this approach [2,3,4,5,6,7].

The motivation to search for wheelchair components that could effectively reduce whole-body vibration originated from a survey conducted as part of a PhD thesis [8]. The survey involved long-term wheelchair users (minimum 1 year of use). More than 80% of respondents reported perceiving vibrations mainly through the seat of the wheelchair, describing them as bothersome and actively seeking ways to minimize them. The studies presented in Chwalik-Pilszyk et al. [5] confirmed that the polyurethane cushion effectively damps vibrations in the 10–40 Hz frequency range. Increasing the thickness of the cushion does not, however, provide additional damping benefits. This is encouraging, as vibrations within this frequency band correspond to the resonance frequencies of the spine and some internal organs, meaning that even a standard cushion thickness can offer protection in the most critical range [9]. To assess the exposure of people with disabilities to whole-body vibration, ISO 2631 [10] is commonly applied. This standard is primarily intended for evaluating vibration exposure in occupational settings. However, research has shown that during wheelchair mobility, the comfort limit—and in some cases even the annoyance threshold—defined in this standard is exceeded [5,8,11].

The mathematical model presented in the article can be applied to virtually any type of wheelchair, provided that the wheelchair-specific parameters are experimentally identified beforehand. In our study, this identification was performed using an electrohydraulic vibration exciter platform (HECKERT pz25) with a static load capacity of 25 kN and an operating frequency range of 1–100 Hz. The tested wheelchair (Unix Breezy Sunrix Medical, 18 kg) was loaded with a 50 kg mass to simulate the force generated by an average user. Harmonic excitations were applied at frequencies of 2, 5, 10, 15, and 20 Hz. Acceleration signals were measured using piezoelectric accelerometers (PCB PIEZOTRONICS 356B18 and 393B31) and analyzed in both the time and frequency domains with Catman Easy AP software. Based on these measurements, the stiffness coefficient (c_0_) and damping coefficient (b_0_) describing the wheelchair–ground interface were determined experimentally. These parameters were then incorporated into the Voigt–Kelvin element of the model. This approach makes it possible to adapt the model to different wheelchair designs simply by repeating the parameter identification procedure. In addition, the entire model was experimentally verified on the same electrohydraulic vibration exciter platform. During the measurements at different excitation frequencies, accelerometers were placed on the head, chest, and pelvic region of a person sitting in the wheelchair. The results indicated that the model provides a reliable representation of vibration transmission to the human body.

We fully acknowledge the beneficial effects of controlled whole-body vibration (WBV) in specific therapeutic applications, such as the treatment of osteoporosis, where exposure occurs at well-defined frequencies and for short durations (e.g., less than 1 h per week). There is evidence that controlled WBV—particularly low-magnitude, high-frequency (LMHF) stimulation—can have positive effects on bone and muscle mass and may help reduce fracture risk in certain populations. However, findings are not entirely consistent: meta-analyses indicate considerable variability in the outcomes, depending on the applied protocol (the phase, magnitude, frequency, and duration of the intervention) as well as the characteristics of the study population [12,13,14,15].

However, there is a fundamental difference between controlled therapeutic WBV and the vibrations experienced by wheelchair users during daily mobility. In systemic vibratory therapy, the amplitude, frequency, and duration of the stimulus are precisely defined and individually adjusted to achieve specific biological effects in a safe and repeatable manner. In contrast, wheelchair users are exposed to uncontrolled, random, and often impulsive vibrations, which depend on surface irregularities, speed, and environmental conditions [11,16,17]. These vibrations can involve broad frequency bands, higher amplitudes, and longer cumulative exposure times. Importantly, wheelchair users also experience sudden jerks (e.g., when crossing curbs or obstacles), which can generate high peak accelerations and pose a potentially harmful mechanical load on the body.

This clear distinction shows why the same physical phenomenon—vibration—may have positive effects in a controlled therapeutic context, but may be detrimental under uncontrolled real-world conditions.

We also find your suggestion regarding the potential active use of polyurethane cushions for delivering controlled vibratory stimulation to be highly valuable and innovative. Although the present study focuses on the passive damping properties of polyurethane, we consider the development of cushions capable of generating predefined vibration profiles for therapeutic applications to be a very promising direction for future research.

Once again, we would like to thank you for your constructive comments, which enrich the discussion and open new research perspectives.

## Data Availability

The original contributions presented in the study are included in the article, further inquiries can be directed to the corresponding author.
